# Acknowledgement to Reviewers of the *Journal of Personalized Medicine* in 2014

**DOI:** 10.3390/jpm5010001

**Published:** 2015-01-08

**Authors:** Journal of Personalized Medicine Editorial Office

**Affiliations:** MDPI AG, Klybeckstrasse 64, CH-4057 Basel, Switzerland

The editors of the *Journal of Personalized Medicine* would like to express their sincere gratitude to the following reviewers for assessing manuscripts in 2014:

Ashworth, Mark
Aylsworth, Arthur S.
Baños, Rosa M.
Berwick, Marianne
Björck, Erik
Bock, Beth
Bohil, Corey J.
Bonham, Vence L.
Boulos, Dina N Kamel
Boulos, Maged N. Kamel
Brown, Sandra
Caini, Saverio
Canestaro, William
Chang, Vivian Y.
Chaudhuri, Shomir
Chen, Lei-Shih
Clayton, Ellen Wright
Cohen, Wendy
del Gaudio, Daniela
Distefano, Johanna K.
Doerr, Megan
Drozek, David
Dunne, Cody
Emmert, Steffen
Friemuth, Robert
Gerber, Lynn H.
Harris, Jennifer R.
Haspinger, Eva R.
Higashi, Takahiro
Huijg, Johanna M.
Remon, J.
Kampel, Martin
Kaufman, David
Keogh, Louise A.
Kim, Yunjung
Kort, Helianthe
Krypotos, Angelos-Miltiadis
Kumar-Sinha, Chandan
Lan, Yuan-Tzu
Langford, Aisha T.
Letra, Ariadne M.
Levine, Jennifer M.
Liotta, Lance A.
Liu, Chunyu
López-Fernández, Luis A.
Manolio, Teri A.
Martínez-Pérez, Borja
Mayer, Deborah K.
McCarty, Catherine A
Meyer, Henriette E.
Molster, Caron
Morris, Alan H.
Nekhlyudov, Larissa
Nielsen, Jeppe Agger
O’doherty, Kieran C.
Ossa, Diego
Overbey, Casey
Palos, Guadalupe
Patrick, K.
Perri, Francesco
Piller, Neil
Postma, Marteen
Postmus, Iris
Powell, Charles
Prochniak, Carrie F.
Pujol, P.
Ragia, Georgia
Redfern, Julie
Redmond, Michelle
Roth, Jacquelyn J.
Sacristán, José A
Schleidgen, Sebastian
Schneider, Susan M.
Solomon, Benjamin
Street, Jackie
Swendeman, Dallas
Tantisira, Kelan G
Tatara, Naoe
Thielke, Stephen
Tomiak, E.
Trosman, Julia
Tuot, Delphine
Van Allen, Eliezer M.
Victorson, David
Vila, Joan
Wasson, Katherine
Weng, Liming
Williams, Marc
Wong, Stuart J.
Yang, Tony
Yen, Tina W. F.

